# Atypical Facial Expressivity in Young Children with Problematic Peer Relationships

**DOI:** 10.1007/s10578-022-01445-1

**Published:** 2022-09-26

**Authors:** Holly Howe-Davies, Antony S. R. Manstead, Stephanie H. M. van Goozen

**Affiliations:** 1 School of Psychology, Cardiff University, Cardiff, Wales, UK; 2 Department of Clinical Child and Adolescent Studies, Leiden University, Leiden, The Netherlands

**Keywords:** Mimicry, Motor empathy, Emotion recognition, Peer problems

## Abstract

Peer problems are frequently associated with difficulties in recognizing and appraising the emotions of others. It has been argued that facial responsiveness to others’ emotions—or motor empathy—is a precursor of emotion processing and affective empathy. Although mimicry impairments have been observed in studies of young people with conduct problems, to our knowledge no study has examined facial responsiveness to others’ expressions in young children and examined how this relates to peer relationship problems. Four- to 7-year-old children (n = 91) with or without teacher-reported peer relationship problems (Strength and Difficulties Questionnaire) viewed three dynamic film clips depicting a sad, happy, or scared child, while their spontaneous facial emotional responses were assessed using iMotions software that codes the movement of facial muscles. Children displayed facial expressivity that was congruent with the emotional expressions in the clips. Groups with and without peer problems did not differ in their responses to seeing a happy child. However, children with peer problems exhibited reduced or atypical facial emotional responses to the negative emotional clips. Decreased or atypical facial expressivity to negative emotions was also associated with severity of peer problems; atypical facial responsivity to sadness and reduced facial responsivity to fear predicted peer problems independently of one another. We conclude that reduced or atypical facial expressiveness in response to other children’s dynamic facial expressions is associated with problematic peer relations in young children. The implications for early identification and interventions to support prosocial development are discussed.

**Supplementary Information:**

The online version contains supplementary material available at 10.1007/s10578-022-01445-1.

## Introduction

Most children exhibit conduct problems from time to time. Typically developing children occasionally fight and intimidate other children, disobey adults, or tell lies. However, when these behaviors extend beyond occasional occurrences, they may adversely affect a child’s socio-emotional and educational development, and lead to other negative outcomes later in life, such as psychiatric illness, poor physical health, violent relationships and erratic employment patterns [1, 2].

Peer problems such as rejection in early childhood are associated with psychological maladjustment and mental health impairments that can continue throughout development [3, 4]. Impaired emotion processing may be responsible for the association between childhood peer problems and psychopathology, the reasoning being that if children cannot correctly identify the emotions of others, they are more likely to respond to others in an inappropriate way [5]. Indeed, difficulties in recognizing, understanding, and interpreting the emotions of others have frequently been associated with peer rejection and exclusion [6] and a range of other psychological problems: social anxiety, psychopathy and autism symptoms are all associated with or mediated by emotion processing abilities [7, 8]. Emotion processing skills at primary-school age are positive predictors of later social and emotional competence [9].

It has been proposed that rapid (short latency) mimicry responses are automatic or preconscious and therefore difficult to control voluntarily, and that they contribute to emotion recognition and may be crucial in the development of empathy [10, 11]. Similarly, the Perception Action Model (PAM; [12]) hypothesizes that observing others’ emotions activates neural circuits, including the amygdala, evoking an emotional state congruent with that of the target person. This facilitates emotion recognition [13] and perspective-taking (cognitive empathy) and supports the development of appropriate social behavior [14], although there are conditions, for example when anger is expressed, in which emotional mimicry is not always socially adaptive [15]. Emotion recognition, in turn, is likely to facilitate mimicry. Problems in facial responding—or motor mimicry—early in life might impair a child’s prosocial development, in particular their ability to recognize and understand others’ emotions and to form friendly, reciprocal relationships with others.

### Facial Mimicry in Children

Studies using facial electromyography (EMG) paradigms to assess facial mimicry have consistently demonstrated that children react with increased *zygomaticus major* activation (lip corner puller; pulling the corners of the mouth upwards) when exposed to happy faces, whereas observing others’ angry faces elicits an increase in *corrugator* muscle activation (brow lowerer; drawing the brows down and together; [16, 17]). There is also some evidence of specific facial muscle response patterns for other emotions. In typically developing (TD) children, aged 6–7 years, Deschamps and colleagues [16, 18] found that sad faces were related to a combination of *corrugator*, *depressor* (lip corner depressor; pulling the corners of mouth downwards) and *frontalis* (inner brow raiser; raising inner brows) activation, and that fearful faces were related to *frontalis* activation.

EMG measurement of facial activity is relatively intrusive, and observational coding of facial behavior is highly demanding. Software solutions have been developed to code the movement of facial muscles automatically, removing the need to attach multiple electrodes to the face. For example, iMotions (2016) includes a facial expression analysis engine called Affectiva AFFDEX, which is grounded in the Facial Affect Coding System (FACS; [19]) and enables the analysis of live or recorded faces [20]. One study using automated computerized coding of facial emotional expressions in students showed that there was less facial mimicry in response to others’ negative facial expressions in those high in psychopathic traits [21]. It has been shown that the Affectiva AFFDEX software can identify facial expressions and that results using its output are comparable to those using EMG [22]. The program also reliably detects happiness, sadness, anger, and fear expressions in young children [23].

### Facial Mimicry and Atypical Social Skills

Studies investigating how facial expressiveness or motor empathy relates to peer problems have focused primarily on older children and young people with conduct problems (CP), due to their significant impairments in peer relations. De Wied and colleagues [17, 24] compared facial EMG responses in boys with disruptive behavior disorder (DBD) and TD boys aged between 8 and 12 years (only boys were included because DBD is more prevalent among boys than girls). When exposed to dynamic facial expressions, both groups showed increased *zygomaticus* activity to happy faces and increased *corrugator* response to angry facial expressions, but boys with CP showed a significantly smaller increase in *corrugator* response to anger [20] and sadness [19]. These results suggest that mimicry responses to negatively valenced facial expressions are less intense in boys with CP.

Further evidence of possible mimicry impairments in children with peer relationship problems comes from emotion recognition studies. Facial emotion recognition deficits, specifically impaired emotion recognition for negative emotions but unimpaired emotion recognition for positive emotions, have been found in children and adolescents with conduct disorder [2, 25].

It is unclear when atypical responses to facial expressions emerge. Deschamps and colleagues [16] investigated EMG activity in response to posed emotional faces in 6- to 7-year-old children, some TD and some with CP or attention deficit hyperactivity disorder (ADHD). They found that all groups displayed significant facial mimicry in response to the emotional expressions, and no differences in mimicry response between the groups. They concluded that mimicry may not be impaired until later in development. However, if motor mimicry plays a key role in the development of emotion recognition and empathy, and if impairments in these emotion processing skills can be observed at a young age [26], it would be reasonable to expect impaired facial emotional expressiveness also to be present from a young age. A potential reason for the lack of significant findings in this study is that researchers used black and white posed facial expressions to assess mimicry. Posed emotional stimuli are less representative of everyday facial expressions and human interactions, and weaker in terms of eliciting mimicry [27].

In the current study we used a validated set of dynamic empathy-eliciting film clips portraying natural expressions of children in real-life settings. The objective was to compare facial expressiveness in 4- to 7-year-old children who did or did not have problematic peer problems as rated by their teachers. Facial responsivity during exposure to the emotional film clips was assessed using the Affectiva AFFDEX facial expression recognition engine. We predicted that children with peer problems would have reduced facial expressivity to negative emotions, and that impairments in facial expressivity for negative emotions would be associated with the severity of peer problems.

## Method

### Participants

Participants (n = 91; 23 girls) were aged between 4 and 7 years and had been referred by their teachers to the Neurodevelopment Assessment Unit (NDAU; (http://www.cardiff.ac.uk/research/explore/research-units/neurodevelopment-assessment-unit) for assessment. Children with emerging neurodevelopmental and mental health problems frequently do not receive the support they need, and even when they do it is often not targeted to their individual needs. The NDAU was set up to assess children who have emerging difficulties at school but have not accessed mental health services. The NDAU has adopted a transdiagnostic RDoC-informed approach (https://www.nimh.nih.gov/research-priorities/rdoc/index.shtml) to assessment, concentrating on cognitive, socio-emotional, and behavioral dimensions. The assessment outcomes are used to generate a report that profiles the child’s strengths and needs and informs the formulation of tailored interventions that are delivered by school staff. This helps those who work with the child at school or home to understand the nature of the child’s difficulties, to select appropriate educational provision, to prioritize interventions and inform later referrals to clinical services.

The NDAU is available to schools that have concerns about a pupil’s functioning and the sample therefore demonstrated a heterogeneous range of difficulties including children with varying levels of emotional and/or behavioral problems. None of the children had received a diagnosis at the time of testing.

As part of the NDAU referral process, the children’s teachers complete the Strengths and Difficulties Questionnaire (SDQ; 28). In the current study, the high peer problems group was selected based on the established norm scores of the SDQ scoring guidelines (retrieved from http://www.sdqinfo.org/py/sdqinfo/b3.py?language=Englishqz(UK)). Forty-five children (32 boys) who had a score of 5 or more on the peer problems subscale of the SDQ were classified as being at high/very high risk of peer problems. The low peer problems group consisted of 46 children (35 boys) who had a score of 4 or less on the peer problems subscale, indicative of having a close-to-average or only slightly elevated risk of peer problems [28]. Analysis of the items on the peer problems subscale showed that participants in the high peer problems group were more likely to be rated as ‘not liked by other kids,’ more likely to ‘play alone,’ and less likely to ‘have at least one good friend’ than children in the low peer problems group.

## Materials

### Strengths and Difficulties Questionnaire (SDQ)

The teacher version of the Strengths and Difficulties Questionnaire (SDQ) is a behavioral screening questionnaire for children and young people aged 3–16 years. Teachers are asked to rate 25 items on a 3-point Likert scale based on how true they are of the child’s behavior over the last 6 months (0 = not true; 1 = somewhat true; 2 = certainly true). The 25 items comprise five subscales; four subscales assess negative behaviors (emotional symptoms, conduct problems, hyperactivity/inattention and peer relationship problems) and one subscale assesses positive behavior (prosocial behavior). In addition to subscale scores, the SDQ also provides a Total difficulties score, comprising all negative behavior scale scores. This is indicative of the severity of psychosocial difficulties a child presents with. The SDQ discriminates well between children with and without psychological problems [29] and is a proven screening tool for child psychiatric disorders in community samples [30]. Based on a large UK community sample, mean teacher-reported scores for both the total and subscale measures are as follows: Total = 6.6; emotional = 1.4; conduct = 0.9; hyperactivity = 2.9; peer = 1.4; prosocial = 7.2 (retrieved from http://www.sdqinfo.org/norms/UKNorm1.pdf).

The peer subscale scores were used to classify children into high or low peer problem groups because elevated scores on the peer problems subscale are reflective of the interpersonal problems displayed by individuals with behavioral problems [31]. The peer problems subscale consists of 5 items assessing social competence, and total scores range from 0 to 10. It has previously been demonstrated that the teacher SDQ peer problems subscale shows an acceptable level of reliability in children aged 4–7 [32].

### Intellectual Ability

The Lucid Ability assessment was administered to measure cognitive ability [33]. The validity of this test is comparable to a range of conventional IQ measures, including the Wechsler Intelligence Scale for Children (WISC-III), the British Ability Scales (Second Edition) and the British Picture Vocabulary Scale (Second Edition; Lucid Ability Administrator’s Manual, 2015; 33).

### Measurement of Facial Responsivity

Children were shown three empathy-inducing film clips that were validated in 3-year-olds [34]. One clip presents child happiness (a boy opening a Christmas present), another sadness (a boy flushing his dead goldfish down the toilet), and another fear (a girl scared of being in a car wash). The film clips varied in length between 52 and 56 s. To avoid two negative videos being presented successively, the happiness video was always shown second, between the sadness and fear videos. Facial behaviors of participants from stimulus onset until stimulus offset were recorded with a Dell laptop (Precision 7710) integrated webcam. The section of each clip in which the child protagonist expressed maximum facial expressions of happiness, sadness or fear was used as the target scene; neutral sections at the start of each clip were used as a baseline. Webcam footage of the participant viewing the target and baseline sections of the clip were imported into iMotions Biometric Research Platform 6.0 software (www.imotions.com) and processed using the Affectiva AFFDEX facial expression recognition engine. The software identifies the main landmarks on the face, such as the eyes and mouth, and assesses movement, shape, and texture of the face at a pixel level. The outputs include measures of activity at seven muscle sites and expression channels (*zygomaticus, corrugator, frontalis, depressor, risorius, orbicularis oculi,* and eye widen). The target scenes varied in length between 9 and 15 s. Baseline sections varied between 8 and 10 s.

### Data Processing

iMotions provides probability-like values for all basic emotions and the muscle sites associated with each emotion. Mean facial probability values for the seven muscle sites mentioned above were calculated for the baseline and target periods of each clip and each child. Trials containing less than 50% data were excluded prior to data analysis, leading to the removal of 11 participants, and scores more than 3 standard deviations from the mean for a variable were replaced with the highest/lowest value within 2 standard deviations of the mean; this applied to approximately 10% of the data. Half of the children (*n* = 46) in the current analyses did not have complete facial responsivity data for all six target and baseline sections (due to difficulties sitting still, a lack of engagement, etc.). There were no significant differences in subscale or total SDQ scores between the group with complete data and the group with missing data. Consequently, all participants were retained in the analyses and the pairwise deletion procedure was employed for missing values.

### Statistical Analysis

To address the first hypothesis, mixed model analyses of variance (ANOVAs) were conducted to examine whether changes in facial activation from baseline differed in children with high peer problems compared to children with low peer problems. Dependent variables were the seven facial mimicry muscle sites in response to each emotion. Responsivity was assessed by the within-subjects factor epoch with two levels (baseline and target). Group was a between-subjects variable with two levels (high peer problems and low peer problems). To address the second hypothesis, Pearson’s correlations examined relationships between key facial responsivity variables and severity of peer problems and a multiple regression analysis was used to determine whether reduced facial responsivity predicted problems with peer relations. A significance level of 0.05 was used in all tests.

## Results

### Preliminary Analyses

Descriptive characteristics of the high and low peer problems groups are shown in Table 1. The groups did not differ in age, gender, ethnicity, socioeconomic indicators, or estimated full-scale IQ (FSIQ). Analyses of the SDQ subscales and total score revealed differences between groups in peer, prosocial and total SDQ scores. The high peer problems group did not differ from the low peer problems group in terms of emotional, hyperactive or conduct problems.

**Table 1:** Descriptive data for children in low and high peer problems groups

	Low peer problems (n = 45)		High peer problems (n = 46)		Low vs High	
	M	SD	M	SD	Statistic	p
Age (month)	76.36	10.77	72.17	13.83	*t*(89) = 1.605	0.112
Gender (% males)	71.1%		77.8%		*Χ*^2^(1) = 0.526	0.468
Ethnicity (% British)	91.1%		82.6%		*Χ*^2^(8) = 9.104	0.334
Socioeconomic indicators						
Parental education (% university degree)	17.8%		17.4%		*Χ*^2^(5) = 2.754	0.738
Income (% less than £20,000 pa)	46.7%		37.0%		*Χ*^2^(7) = 8.976	0.254
FSIQ	98.71	11.40	101.02	12.14	t(85) = − 0.909	0.336
SDQ Emotional	4.40	2.38	3.98	2.39	t(89) = 0.844	0.401
SDQ Hyperactive	8.69	1.56	8.24	1.73	t(89) = 1.301	0.197
SDQ Conduct	4.84	2.31	4.39	2.26	t(89) = 0.948	0.346
SDQ Peer	2.69	1.18	5.91	1.01	t(89) = − 14.005	< 0.001*
SDQ Prosocial	3.80	2.39	2.57	2.34	t(89) = 2.488	0.015*
SDQ Total	20.62	3.18	22.52	4.38	t(89) = − 2.373	0.020*

*FSIQ* Full Scale Intelligence Quotient; *SDQ* Strengths and Difficulties Questionnaire

Given that children with peer problems also differed in prosocial behavior, the relation between the two subscales was further investigated. Scores on the peer problems subscale were significantly inversely associated with prosocial scores (*r* = − 0.325, p < 0.01). Although scores on the peer problems subscale were significantly correlated with key responsivity measures, the same was not true for the low prosocial scores (see Supplementary Table). In addition, combined peer problems and reversed prosocial scores did not correlate with key responsivity measures (all *p* values > 0.05).

Prior to conducting the main analyses, baseline facial responsivity scores were subjected to independent-samples *t*-tests comparing children who were high and low in peer problems. No significant differences emerged on any measure (all *p*s > 0.05).

## Main Analyses

### Facial Responsivity in Children with High vs Low Peer Problems

**Fig. 1:**
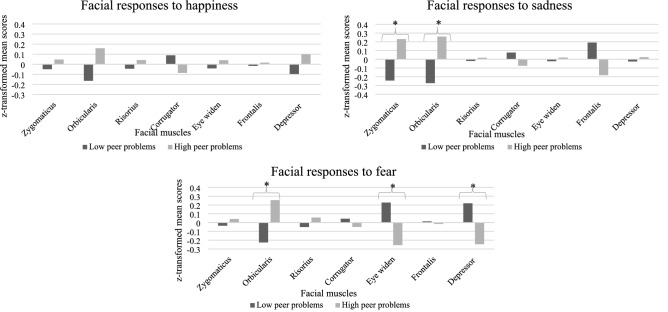
Children’s facial expressivity in response to another child’s facial happiness, sadness, and fear as a function of peer problems group. For visualization purposes standardized mean scores (z scores) are shown. Happiness/Low peer, *n* = 36. Happiness/High peer, *n* = 37. Sadness /Low peer, *n* = 39. Sadness/High peer, *n* = 41. Fear/Low peer, *n* = 36. Fear/High peer, *n* = 32

We assessed whether there were differences in facial responsivity between groups with high and low peer problems, and univariate analyses assessed changes from baseline. As detailed below, there were group differences in mean facial responsivity for the happiness, sadness, and fear clips. These group differences in facial responsivity during stimulus presentation are illustrated in Fig. 1, which shows standardized mean scores (mean facial responsivity scores transformed into z-scores).

There were statistically significant main effects of epoch in response to happiness. Specifically, happy faces elicited the expected increase in *zygomaticus*, *F*(1, 68) = 5.499, *p* = 0.022, ηp^2^ = 0.06, and *frontalis* activity, *F*(1, 68) = 4.852, *p* = 0.031, ηp^2^ = 0.07, and a decrease in *orbicularis oculi* activity, *F*(1, 68) = 17.413, *p* < 0.001, ηp^2^ = 0.20, relative to baseline (see Table 2). There were no main effects of group, and nor were there any epoch by group interactions, indicating that children with high or low peer problems did not differ in facial responsivity to happiness.

**Table 2:** Key facial mimicry muscle site activation during emotional clips compared to baseline activity

Emotional clip	Baseline		Target emotion	
Muscle site	M	SD	M	SD
Happiness				
Zygomaticus	12.52	19.72	18.97	26.01
Frontalis	6.55	8.49	8.22	10.32
Orbicularis oculi	6.34	7.59	3.17	3.40
Sadness				
Frontalis	6.15	7.84	13.61	18.95
Corrugator	0.51	0.94	1.97	3.66
Depressor	0.25	0.60	3.69	6.88
Eye widen	0.11	0.31	0.45	1.36
Fear				
Risorius	1.06	1.90	5.79	10.31
Eye widen	0.20	0.58	0.48	1.08
Frontalis	14.50	17.84	9.88	10.79
Zygomaticus	7.23	13.61	16.99	25.95

*Zygomaticus* lip corner puller; *frontalis* inner brow raiser; *orbicularis oculi* cheek raiser; *corrugator* brow lowerer; *depressor* lip corner depressor; *risorius* lip stretcher. For pictorial examples of the associated facial actions, see https://imotions.com/blog/facial-action-coding-system/#main-action-units. Happiness Baseline, *n* = 74; Happiness Target, *n* = 73; Sadness Baseline, *n* = 77; Sadness Target, *n* = 80; Fear Baseline, *n* = 62; Fear Target, *n* = 68

In response to sadness, the ANOVAs yielded significant main effects of epoch. Sad facial expressions elicited significantly more *frontalis*, *F*(1, 73) = 13.776, *p* < 0.001, ηp^2^ = 0.16, *corrugator*, *F*(1, 73) = 13.597, *p* < 0.001, ηp^2^ = 0.16, *depressor*, *F*(1, 73) = 19.544, *p* < 0.001, ηp^2^ = 0.21, and eye widen activity, *F*(1, 72) = 5.086, *p* = 0.019, ηp^2^ = 0.08, during the stimulus than during baseline (see Table 2). There were no significant main effects of group but, consistent with predictions, there were significant group by epoch interactions. *Zygomaticus*, *F*(1, 73) = 9.803, *p* = 0.003, ηp^2^ = 0.19, and *orbicularis oculi*, *F*(1, 73) = 5.098, *p* = 0.027, ηp^2^ = 0.07, activity was lower during stimulus exposure than during baseline in the low peer problems group, whereas activation at these muscle sites increased in children with high peer problems.

Independent samples *t*-tests showed significantly greater levels of *zygomaticus* and *orbicularis oculi* activity in the high peer problems group compared to the low peer problems group during presentation of the emotive section of the sad clip [*zygomaticus*: *M*_low_ = 9.23, SD = 14.24, *M*_high_ = 19.30, SD = 25.40, *t*(63.5) = − 2.200, *p* = 0.031, *d* = 0.49, 95% CI [− 19.21, -0.93]; *orbicularis oculi*: *M*_low_ = 4.26, SD = 6.70, *M*_high_ = 8.23, SD = 7.44, *t*(78) =  − 2.454, *p* = 0.016, *d* = 0.56, 95% CI [− 7.19, − 0.75]].

There were also significant main effects of epoch in responses to fear. Specifically, seeing a fearful facial expression led to the expected increase in *risorius*, *F*(1, 59) = 14.370, *p* < 0.001, ηp^2^ = 0.20, and eye widen activity, *F*(1, 59) = 4.835, *p* = 0.032, ηp^2^ = 0.08, compared to baseline. However, there was also an unexpected decrease in *frontalis* activity, *F*(1, 59) = 5.721, *p* = 0.020, ηp^2^ = 0.09, and an increase in *zygomaticus* activation, *F*(1 59) = 12.334, *p* = 0.001, ηp^2^ = 0.17 (see Table 2). There was a significant main effect of group for the activation of the *orbicularis oculi*, *F*(1, 59) = 5.931, *p* = 0.018, ηp^2^ = 0.09, and, consistent with predictions, a significant interaction. Eye widen activity increased during exposure to fear in the low peer problems group, but decreased in children with high peer problems, *F*(1, 59) = 5.564, *p* = 0.022, ηp^2^ = 0.09.

Independent samples t-tests revealed significantly less eye widen activity [t(53.5) = 2.110, p = 0.040, d = 0.50, 95% CI [0.03, 1.02], *M*_low_ = 0.72, SD = 1.30, *M*_high_ = 0.20, SD = 0.67], less *depressor* activity [t(54.2) = 2.021, p = 0.048, d = 0.48, 95% CI [0.01, 2.45], *M*_low_ = 2.26, SD = 3.20, *M*_high_ = 1.03, SD = 1.68] but marginally more *orbicularis* activity [t(53.9) =  − 1.982, p = 0.053, d = 0.49, 95% CI [− 11.78, 0.07], *M*_low_ = 6.15, SD = 9.61, *M*_high_ = 12.01, SD = 14.05] in the high peer problems group, compared to the low peer problems group, during the presentation of the emotive section of the fearful clip.

### The Relationship Between Facial Mimicry and Peer Problems

The relation between peer problems and facial responsivity to sad and fear stimuli was further supported by correlational analysis. While watching the sad video, peer problems were positively associated with *orbicularis oculi* activity, *r* = 0.248, *p* < 0.05, negatively associated with *frontalis* activity, *r* = -0.228, *p* < 0.05, and marginally positively associated with *zygomaticus* activity, *r* = 0.214, *p* = 0.057. While watching the fear video, peer problems were significantly inversely related to eye widen activity, *r* = − 0.287, *p* =  < 0.05.

Multiple regression analysis was used to determine whether increased *orbicularis oculi* and *zygomaticus* facial activity during the observation of sadness, decreased *frontalis* activity during the observation of sadness, and reduced eye widen during the observation of fear predicted severity of problems with peer relations. The multiple regression model was significant, *F*(4,59) = 4.23, *p* < 0.01, adjusted *R*^2^ = 0.18. Reduced *frontalis* activity in response to sadness and reduced eye widen activity in response to fear significantly added to the prediction, *p* < 0.05 (see Table 3).

**Table 3:** Summary of multiple regression analysis

Facial measure	B	SEB	β	CI
Intercept	4.354	0.357		
*Frontalis* sad	− 0.025	0.012	− 0.249*	− 0.050, 0.000
*Orbicularis oculi* sad	0.039	0.034	0.149	− 0.028, 0.107
*Zygomaticus* sad	0.023	0.013	0.238	− 0.002, .048
Eye widen fear	− 0.444	0.221	− 0.246*	− 0.887, − 0.001

*B* unstandardized regression coefficient, *SEB* standard error of the coefficient, *β* standardized coefficient, *CI* confidence intervals, * = *p* < .05

## Discussion

Impairments in facial responding have been hypothesized to limit a child’s ability to recognize and understand others’ emotions, and in turn, their ability to form friendly, reciprocal relationships with others [35, 36]. We investigated differences in facial responsiveness and their associations with problematic peer relationships in a sample of children with varying levels of teacher-rated peer problems. Consistent with previous research, our results demonstrate reduced or atypical facial responsiveness to others’ expressions of negative emotions among children with problematic peer relations. We also explored the relationships between key facial responsiveness variables and peer problems. These results show that facial responsiveness to expressions of negative emotions is associated with severity of peer problems. To our knowledge this is the first study to show that reduced *frontalis* activity during the observation of sadness is associated with the severity of problematic peer relations, as rated by teachers.

Before discussing these findings, it is worth noting that the results also provide evidence that, overall, young children were facially responsive to another child’s expressions of happiness, sadness, and fear. Children displayed increased *zygomaticus* and *frontalis* activity and decreased *orbicularis oculi* activity in response to happy expressions. Observing another child’s sad facial expressions led to increases in *corrugator*, *frontalis*, *depressor*, and eye widen activity. Observing fear expressions resulted in an increase in *risorius*, *zygomaticus,* and eye widen activity, and a decrease in *frontalis* activation. Several of these changes in facial activity can reasonably be regarded as evidence of motor mimicry (e.g., increased *zygomaticus* activity to happy expressions; increased *corrugator* and *frontalis* activity to sad expressions, and increased eye widen activity to fear expressions). Other changes in expressiveness are less obviously reflections of the stimulus expressions (e.g., decreased *orbicularis oculi* activity to happy expressions, and increased *zygomaticus* activity to fear expressions) but may reflect a mixed or ambivalent emotional response.

The current study is to our knowledge the first to use automated software to assess facial expressiveness in young children and to link it to problems in social functioning. It is worth briefly considering some of the advantages and disadvantages of using automated facial expression analysis. An obvious advantage is that it is considerably more efficient in terms of time needed to learn and use the scoring method than its most obvious alternative, the Facial Action Coding System [19]. A further advantage is that the results are more reliable in the sense that a given expression will always by scored the same way by an automated analysis system, whereas human observers are subject to possible effects of fatigue and coding bias. Nevertheless, questions can be raised about the validity of automated analysis. Although studies comparing automated and manual scoring of facial expressions generally show high rates of agreement (e.g., [37, 38]) there is a dearth of evidence relating specifically to children’s expressions. It is also possible that human observers can detect subtle facial movements that are missed by an automatic system, especially if lighting conditions are sub-optimal.

Several differences between children with and without problematic peer relations were observed. In response to sad faces, whereas reduced *zygomaticus* activity was observed in children without problematic peer relations, there was an increase in *zygomaticus* activity in those with impaired peer problems. Moreover, *zygomaticus* and *orbicularis oculi* activation were significantly greater in children with peer problems than in those without such problems. Thus, children with problematic peer relations exhibited an expression incongruent with the sadness expressed by the other child. The absence of a congruent response to sad expressions suggests that children with problematic peer relationships respond atypically to expressed sadness, possibly reflecting a lack of concern about the distress that the other child is expressing.

Children with peer problems also displayed a pattern of facial activation in response to another child’s expression of fear that differed from the one shown by the children without peer problems. In the low peer problems group, the fearful clip significantly enhanced eye widen activity, but did not do so in the high peer problems group; moreover, eye widen activity was significantly greater amongst children without peer problems. Children without peer problems also exhibited significantly more *depressor* activity and significantly less activation of the *orbicularis oculi* during this clip, compared to children with peer problems. These findings suggest children with problematic peer relations did not respond to another child’s expression of fear with a pattern of facial activation congruent with fear. As was the case with sadness, the absence of a congruent response to fear expressions suggests that these children do not share the emotion of the child in the clip.

Correlational analysis provided further support for the view that problematic peer relations are associated with distinctive patterns of facial responsiveness to another child’s emotional expressions. Extent of peer problems was associated with more *orbicularis oculi* and *zygomaticus* activity and less *frontalis* activity in response to sadness, and less eye widen activity in response to fear. Previous research has consistently demonstrated that difficulties in recognizing and appraising the emotions of others is associated with problematic peer relations (e.g., 6). The current results extend these findings by demonstrating that incongruent or inappropriate facial responses to another child’s emotions are associated with poorer peer relations.

Regarding facial responsivity to happiness, our hypothesis that there would be no difference between the two groups was confirmed: Children with problematic peer relations exhibited impaired facial responsiveness to negative but not positive emotions. This is consistent with the findings reported by de Wied and colleagues [17, 24], who found specific facial mimicry deficits for negatively valenced emotions in older children with diagnosed conduct problems. These results highlight the importance of early detection of at-risk children. The children in our sample were exhibiting behaviour that made them at high risk of future psychopathology. Thus, their impairments in facial responsiveness to the emotions of others are similar to those reported in older children who have already been diagnosed and are receiving support from mental health services. It has been proposed that these specific impairments contribute to emotion recognition and are crucial in the development of empathy [10, 11]. In turn, difficulties in recognizing, understanding, and interpreting the emotions of others have frequently been associated with a range of psychological problems, including social anxiety, psychopathy and autism [7, 8]. Future research should expand on the research conducted here by further exploring the associations between facial mimicry, emotional recognition and empathy in young children who exhibit problematic social interactions. Understanding how risk factors interact may help with the early identification of children at risk of psychopathology and enable them to receive help and support at the earliest point.

To our knowledge this is the first study to demonstrate facial responsiveness deficits in children as young as 4–7 years. A previous study of facial mimicry in 6- to 7-year-old children with disruptive behavior disorder and TD controls failed to find group differences in facial responses [16]. This led the authors to conclude that mimicry is unimpaired until later in development. Our study points to a different conclusion. There are numerous possible explanations for the inconsistency in results. First, Deschamps and colleagues [16] used facial EMG techniques, whereas we used the Affectiva AFFDEX facial expression recognition engine. Although these different measurement systems produce broadly comparable results, there are also some differences [22]. Second, Deschamps et al. used stimuli consisting of black and white dynamic pictures of faces, the present study used full color film clips showing children experiencing real-life emotions and the target emotion was displayed in a social context familiar to young children. Thus, there are reasons for thinking that the current procedure was more ecologically valid and may also have been better able to elicit facial expressions in young children.

Despite these strengths, it is also important to acknowledge there are limitations. First, the software used to code young children’s facial behavior may be less valid than alternative measurement systems. Second, all participants were referred to our center for a range of emotional and behavioral problems. Although participants in the low peer problems group had low levels of peer problems, they had elevated levels of conduct and hyperactivity problems and low levels of prosocial behavior; it is therefore worth noting that the low peer problems group is not representative of 4- to 7-year-old children more broadly. Third, teachers’ ratings were used to identify the extent of peer problems in our sample. Although teachers are well placed to observe how children interact with their peers at school, triangulating their ratings with other sources of evidence would yield a more reliable measure. Finally, our study is cross-sectional in design, with the result that we are unable to draw conclusions about the nature and direction of any possible causal relation between problematic peer relationships and facial expressiveness in response to other children’s emotional expressions.

In summary, using automated facial analysis software we observed predicted differences in facial responsiveness to emotional expressions between children with and without problematic peer relations. Reduced or atypical facial responsivity to others’ facial expressions may be associated with other difficulties reported in children with social impairments, such as poor emotion recognition, and reduced cognitive and affective empathy [25, 34]. The results have implications for the development of interventions that focus on improving emotion processing in children who have problematic peer relations. This type of intervention has been shown to be effective. For example, emotion training programs for children and young people have been associated with improved behavior and prosocial skills [39, 40]. Offering emotion interventions at a time when children are still in the process of developing the socio-emotional and cognitive skills that are important for wellbeing and resilience can help to promote prosocial development and protect children from developing more serious mental health problems later in life [2].

## Summary

Empathy, a key skill in children’s socio-emotional development, is initiated by observing another’s emotional state, which often elicits the same expression in the observer. Although behavioral problems, especially problematic peer relationship problems, have been linked to difficulties in recognising and appraising emotions in others, facial expressivity in response to another child’s emotions has not previously been studied in young children with emerging socio-emotional problems. We examined facial emotional expressivity using advanced facial analysis software in 91 4- to 7-year-old children who were referred by teachers because of behavioral problems at school. Children with significant teacher-reported peer problems exhibited facial expressivity that was decreased or incongruent with sad and fearful emotional expressions shown by children of similar age. Dimensional analyses further revealed that decreased facial expressivity was associated with severity of peer problems, regardless of emotion type. Because expressivity in response to another’s emotion plays a role in the development of empathy, these findings have important implications both for early identification of those who are more vulnerable and for support of prosocial development more broadly.

### Supplementary Information

Below is the link to the electronic supplementary material.

Supplementary file1 (XLSX 14 KB)
